# Appropriate timing of performing abdominal ultrasonography and termination of follow-up observation for antenatal grade 1 or 2 hydronephrosis

**DOI:** 10.1186/s12894-020-00750-y

**Published:** 2020-11-03

**Authors:** Akihiro Nakane, Kentaro Mizuno, Taiki Kato, Hidenori Nishio, Hideyuki Kamisawa, Satoshi Kurokawa, Tetsuji Maruyama, Takahiro Yasui, Yutaro Hayashi

**Affiliations:** 1 Education and Research Center for Community Medicine, Nagoya City University Graduate School of Medical Sciences, Nagoya, Japan; 2 Department of Urology, Gamagori City Hospital, Gamagori, Japan; 3 Department of Nephro-urology, Nagoya City University Graduate School of Medical Sciences, Nagoya, Japan; 4 Department of Pediatric Urology, Nagoya City University Graduate School of Medical Sciences, 1 Kawasumi, Mizuho-cho, Mizuho-ku, Nagoya, 467-8601 Japan

**Keywords:** Antenatal hydronephrosis, Ultrasonography, Continuous follow-up period

## Abstract

**Background:**

Most cases of antenatal the Society of Fetal Urology (SFU) grade 1or 2 hydronephrosis (HN) improve or resolve spontaneously with conservative treatment. However, there is no consensus on the duration of follow-up for cases of grade 1or 2 HN. The aim of this study was to determine the need for continuous follow-up period and new management of children with antenatal grade 1or 2 HN.

**Methods:**

Subjects underwent ultrasonographic assessment for HN according to the SFU classification. We retrospectively evaluated 112 patients with postnatal grade 1 HN and 69 with grade 2 HN using abdominal ultrasonography between January 2010 and December 2017. We examined the change in HN grade on repeat ultrasonography. Kaplan–Meier method was used to show the effect of HN grade on the rate of HN changes.

**Results:**

The mean follow-up duration was 44.9 ± 36.4 months (range 12–274). Initial SFU grade 1 HN disappeared in 47.0% of cases at 12 months, 66.4% at 24 months and 73.2% at 48 months. Initial SFU grade 2 HN showed improvement in grade in 74.7% of cases at 12 months, 88.3% at 24 months and 89.5% at 48 months. However, 14.6% of SFU grade 1 and 2.8% of SFU grade 2 cases increased in grade and of the 17 cases, 16 cases worsened within the first 6 months. No cases with increased grade required pyeloplasty. Initial disappearance and later reappearance of HN occurred in 40.5% of SFU grade 1 and 2 cases. The mean duration of later reappearance of HN was 39.1 ± 36.2 months (range 12–137). No cases showed reappearance of HN after more than 1 year.

**Conclusions:**

Ultrasonography within the first 6 months was necessary for management of children with antenatal grade 1or 2 HN, because some patients showed worsening. After that, it is considered safe to spread the follow-up interval for stable cases. Most cases of grade 1or 2 HN resolved spontaneously, however a few cases reappeared within 1 year. Therefore, ultrasonography after 1 year was necessary in children with HN that spontaneously disappeared. The appropriate time to end the follow-up was considered to have been after 1 year or more has passed since the disappearance was confirmed.

## Background

Fetal hydronephrosis (HN) is the most common anomaly detected on antenatal ultrasonography, with an estimated prevalence of approximately 2–5.5% [1–3]. In most cases, HN is diagnosed in the absence of urinary tract obstruction, when the anteroposterior diameter of the renal pelvis is just above the normal range for gestational age [4]. Studies have shown that most cases of antenatal HN improve or resolve spontaneously with conservative treatment [2, 3]. The cases of 98% in the Society of Fetal Urology (SFU) grade 1or 2 HN improve or resolve in a systematic review of seven articles meta-analysis [5]. The resolution rate of SFU grade 1 and 2 are reported to be 98–99% and 60–98% in two prospective studies [6, 7]. However, some patients do not show improvement or increase grade of HN, and a few cases need operation [8, 9]. The detection and postnatal follow-up of persistent antenatal HN is believed to help in the early recognition and prevention of progressive renal damage [3, 10–12]. There is no consensus on the duration of follow-up for cases of grade 1or 2 HN [13]. In patients with SFU grades 1 and 2 HN, a more abbreviated follow-up may be warranted and could save costs [14]. However, It has been pointed out that SFU grade 1 and grade 2 show different natural course [7], and there is concern whether similar follow-up may be acceptable for SFU grade 1 and grade 2. The aim of this study is to examine the hypothesis that there are different continuous follow-up period and new management for children with antenatal SFU grade 1or 2 HN.

## Methods

### Patient characteristics

Ultrasonographic assessments of HN grade were performed according to the SFU classification [15]. We let patients hydrate and test the patient’s bladder when it was not full. All patients visited our institutions from 48 h after birth within 1 month of age and assessed to divide their HN groups as a baseline. We retrospectively evaluated 112 antenatally-detected HN patients with grade 1 unilateral HN and 69 with grade 2 unilateral HN using abdominal ultrasonography at our institutions between January 2010 and December 2017. There were 11 excluded patients who had repeated febrile urinary tract infections or hydroureter and those who were diagnosed with symptomatic vesicoureteral reflux on voiding cystourethrography or diagnosed with ureterovesical junction obstraction on magnetic resonance imaging. Seven cases (2 in grade 1 and 5 in grade 2) had hydroureter and were excluded prior to the initial follow-up of this study. Four cases in grade 2 were excluded during this follow-up due to repeated febrile urinary tract infections. These cases are not added to the total number of each grade. Patient characteristics at baseline were shown in Table 1.

**Table 1:** Patient characteristics at baseline (between 48 h after birth and 1 month of age)

Parameter	Number	Percentage (%)
Total	181	100
Gender		
Male	148	81.8
Female	33	18.1
Laterality		
Right	70	38.7
Left	111	61.3
SFU grade		
Grade 1	112	61.9
Grade 2	69	38.1
Excluded patients		
Total	11	100
Urinary tract infection	5	45.5
Hydroureter	6	54.5

### Follow-up surveillance

The patients were followed up according to the grade of HN, and ultrasonography was performed once every 1–3 months and diagnosed HN of SFU grade by several pediatric urologists. Single pediatric urologists (AN) reviewed all Ultrasonography as a second blinded reviewer and if the first several urologist and the second single reviewer disagreed, the diagnosis of the second reviewer was adopted to minimize inter and intra-rater reliability [16]. In the process of adopting the opinion of the second reviewer, 3 cases in grade 2 were upgraded from grade 2 to 3. The term “resolved” indicated a change in the SFU grade to grade 0, and includes all the final resolutions; “improved” indicated a decrease in the SFU grade at the final evaluation by one or more levels, and this included resolved cases; “no change” indicated no change in the initial SFU grade; and “worsened” indicated an increase in the SFU grade, includes temporary upgrade. In the further follow-ups, repeat ultrasonography was performed for low (1–2) SFU grade HN, while renal nuclear medicine studies using dynamic scanning modes were performed for HN that progressed to a high (3–4) SFU grade [17]. Of the resolved cases, 42 cases were followed up every 1–6 months to examine whether the condition of resolution was stable.

All these procedures were performed after obtaining informed consent for clinical care of follow-ups form the parents. These clinical data were obtained as a retrospective study. Additionally, the procedures were performed as a clinical trial at our institution after obtaining institutional review board approval (approved by Ethical Committee of the Gamagori City Hospital, approval no. Gamabyo 500-4). All statistical analyses were performed using SPSS Statistics Ver. 22 (IBM, Armonk, NY, USA). Values are shown as mean ± SD (range minimum–maximum). Kaplan–Meier method was used to show the effect of HN grade on the rate of HN changes and compared with the use of the log-rank test. All tests were 2-sided. A p value of less than 0.05 was considered statistically significant.

## Results

**Fig. 1:**
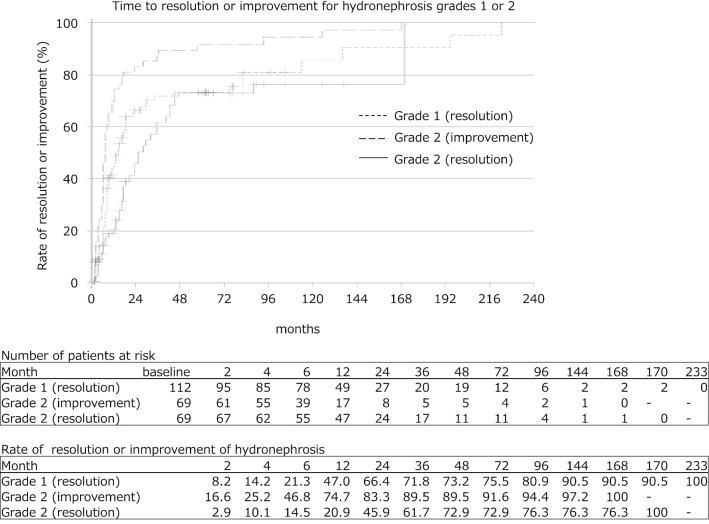
Rate of time to resolution or improvement of hydronephrosis in grade 1 or 2. Kaplan–Meier method. Points displayed as crosses shows censored cases. The upper table in this figure shows the number of affected patients at risk at each observational point. The lower table in this figure shows the rate (%) of resolution or improvement of hydronephrosis at each observational point. The rate of resolution or improvement of hydronephrosis was significantly higher in grade 2 than grade 1 (log-rank test, χ^2^ = 14.679, *P* = 0.00013). However, the rate of resolution of HN was significantly higher in SFU grade 1 than grade 2 (log-rank test, χ^2^ = 3.839, *P* = 0.05)

**Fig. 2:**
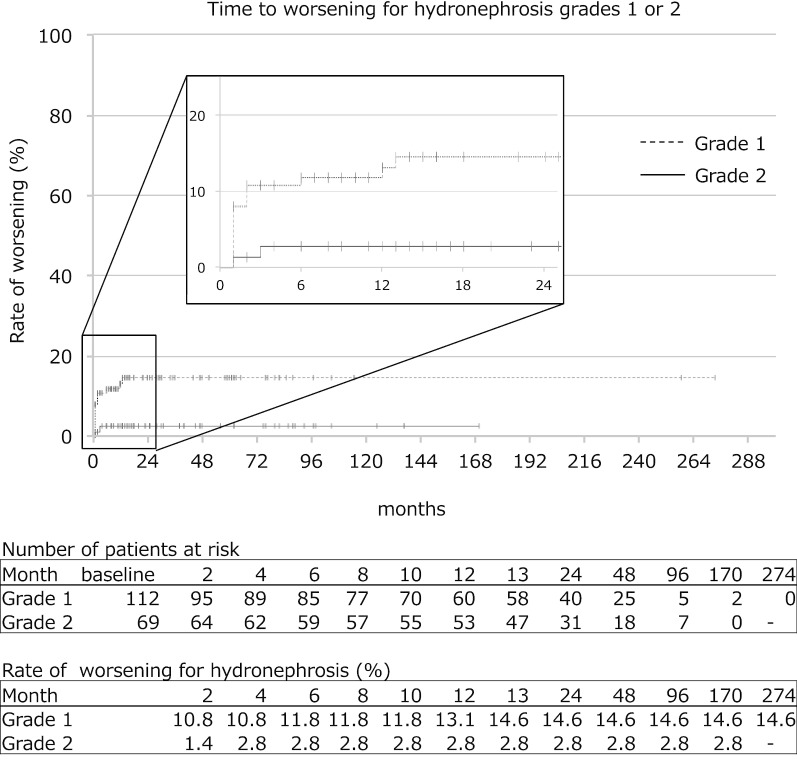
Rate of time to worsening of hydronephrosis in grade 1 or 2. Kaplan–Meier method. Points displayed as crosses shows censored cases. The upper table in this figure shows the number of affected patients at risk at each observational point. The lower table in this figure shows the rate (%) of worsening of hydronephrosis at each observational point. The rate of worsening of hydronephrosis was significantly higher in grade 1 than grade 2 (log-rank test, χ^2^ = 6.227, *P* = 0.013). There were 14.6% of grade 1 and 2.8% of grade 2 cases increased in grade. Then, 99.3% of these cases worsened within the first 6 months, only one case of grade 1 worsened at the 13 months over the study period

**Fig. 3:**
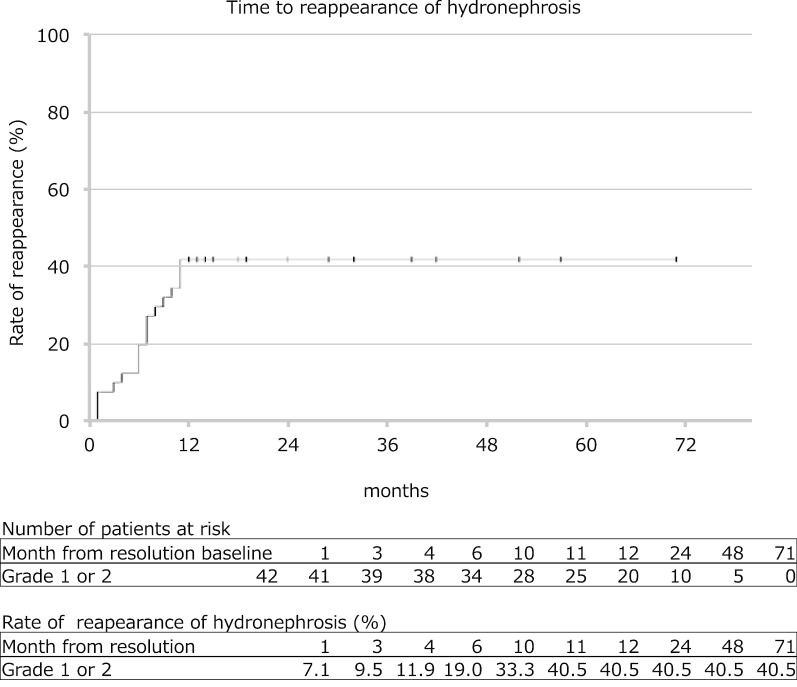
Rate of time to reappearance of hydronephrosis. Kaplan–Meier method. Points displayed as crosses shows censored cases. The upper table in this figure shows the number of affected patients at risk at each observational point. The lower table in this figure shows the rate (%) of reapearance of hydronephrosis at each observational point. No cases showed reappearance of hydronephrosis after more than 1 year

The mean follow-up duration was 44.9 ± 36.4 months (range 12–274). Patients’ characteristics at baseline was shown in the Table 1. The rate of time to resolution or improvement in SFU grade 1 or 2 were determined using Kaplan–Meier method (Fig. 1). Initial SFU grade 1 HN resolved in 47.0% of cases at 12 months, 66.4% at 24 months and 73.2% at 48 months. The median of time to resolution was 14 months (95% confidence interval (CI) 9.49‒18.51). Initial SFU grade 2 HN showed improvement in grade in 74.7% of cases at 12 months, 88.3% at 24 months and 89.5% at 48 months. The median of time to resolution was 7 months (95% CI 6.15‒7.85). Initial SFU grade 2 HN resolved in 20.9% of cases at 12 months, 45.9% at 24 months and 72.9% at 48 months. The median of time to resolution was 25 months (95% CI 14.71‒35.29). The rate of resolution in grade 1 HN or improvement of grade 2 HN was significantly higher in SFU grade 2 than grade 1 (log-rank test, χ^2^ = 14.679, *P* = 0.00013). However, the rate of resolution of HN was significantly higher in SFU grade 1 than grade 2 (log-rank test, χ^2^ = 3.839, *P* = 0.05). On the other hand, 14.6% of SFU grade 1 (15 cases) and 2.8% of SFU grade 2 (2 cases) increased in grade, including cases of temporary upgrade. Of the 17 cases, 16 cases worsened within the first 6 months and only one case of SFU grade 1 showed beyond 6 months (at the 13 months). The rate of time to worsening in SFU grade 1 or 2 were determined using Kaplan–Meier method (Fig. 2). The rate of worsening of HN was significantly higher in SFU grade 1 than grade 2 (log-rank test, χ^2^ = 6.227, *P* = 0.013). None of the patients underwent pyeloplasty due to deterioration of renal function. We followed up on 42 cases that HN resolved. The mean additional follow-up duration was 39.1 ± 36.2 months (range 12–137). Initial disappearance and later reappearance of HN occurred in 40.5% (17 cases). The mean duration of later reappearance of HN was 6.4 ± 3.5 months (range 1–11). The rate of time to reappearance of HN was determined using Kaplan–Meier method (Fig. 3). No cases showed reappearance of HN after more than 1 year. Reappeared HN resoluved again in 10 cases. Reappeared HN of SFU grade 1 or 2 remained in 7 cases. However, none of the patients underwent pyeloplasty during the observation period.

## Discussion

Most cases of antenatal HN improve or resolve spontaneously with conservative treatment [2, 3]. However, some patients do not show improvement without treatment. No consensus currently exists regarding the optimal schedule and duration of follow-up with SFU grade 1 or 2 HN. It is unclear which neonates require postnatal evaluation, when postnatal evaluation for HN should be performed, how long follow-up should be continued, how long an examination should be performed [13].

The timing of postnatal resolution of HN is quite variable, occurring over the first few years of life. Despite variabilities in the underlying diagnoses, mild grades of HN generally show early resolution, with most cases of SFU grade 1–2 HN resolving within 12–18 months of age [8, 18, 19]. In our study, most cases of SFU grade 1–2 HN showed natural improvement or resolution within 4 years of age. In particular, since SFU grade 2 HN resolve more slowly than grade 1 on the early follow-up, the follow-up period is likely to be longer for many cases. However, A small number of cases showed worsening from SFU grade 1 to 2 or SFU grade 2 to 3, and this worsening was mainly noted within 6 months of age in our study. In SFU grade 2, the HN grade that increased within 6 months was a change to the higher risk SFU grade 3, so careful observation may be required for SFU grade 2 during this period. A previous study reported the need for surgical intervention in a small percentage of cases of mild-grade HN [20]. In another previous report, of 225 kidneys with SFU grade 2 HN, 3 showed worsening of HN to a severe grade [8]. Furthermore, following SFU grade 3 cases, there are cases that finally required surgery [21], so it is concluded that more closely monitoring is required than SFU grade 2 [17]. Previous study has been suggested that SFU grade 1 may be more physiologic in nature [22]. If SFU grade 1 persists in perpetuity without need for intervention, perhaps it does not need to be followed as aggressively. We think that 6 months and 1 years SFU grade 1 patients should undergo ultrasonography at the time when the grade of HN may worse. After that, it is considered safe to stop close follow-up and spread the follow-up interval to about 1 year for stable cases. On the other hand, in SFU grade 2 patients, there is a possibility of worsening high grade of HN at 6 months or less, so it is considered necessary to perform serial ultrasonography. However, after that period, it will be possible to spread the follow-up interval as with SFU grade 1.

Operative repair was not required in all cases in our study. Since there were no patients who finally had surgery, it was thought that the follow-up interval could be spreaded. However, our study showed that there is a high probability that HN will reappeare immediately after it disappears. It is considered that reappearance of HN needs to be noted as a new finding against the question of whether it is sufficient to confirm the disappearance of HN once at the end of follow-up. In our study, once HN disappeared for more than 1 year, no case had reappearance of HN. It was suggested that follow-up could be completed if there was no reappearance for more than 1 year. In our study, there were 7 cases that remained recurrent. Although no cases have been operated on, it is not possible at this time to describe what kind of natural history these cases have and new prospective studies are needed to determine its natural history.

Chertin et al. reported that 50% of cases requiring surgical intervention undergo surgery within the first 2 years and almost all cases undergo surgery within the first 4 years [23]. These authors recommended evaluation every 3–6 months during the abovementioned period [23]. Some authors have proposed that further evaluation is unnecessary for SFU grade 1 or 2 [14, 24]. Others have advised serial US until decrease or resolution of HN, or until patients are old enough to communicate symptoms of renal colic [25, 26]. Taken together the above results, we propose the end of follow-up when it can be confirmed that HN disappearance continues after 1 year, or when low grade of HN remains stable for more than 4 years (enough to communicate symptoms) while the follow-up interval is spreaded.

Our study had some limitations. This was a retrospective study. Additionally, follow-up indications were not standardized across the participating physicians. The evaluation grade of HN was SFU classification only, anterior–posterior diameter measurement [27] and UTD classification [28] are not performed. Furthermore, this cohort may have not only ureteropelvic junction obstructon but also other complicating malformations, including asymptimatic vesicoureteral reflux, because we do not routeinly do voiding cystourethrography or magnetic resonance imaging on asymptomatic HN patients. SFU grading system has problems with inter and intra-rater reliability [16, 29]. We tried to minimize the problems by adopting a second single blinded reviewer to minimize bias from the treating physician, however the other problem remains that reliability for grade 2 and 3 is rather low [16, 30]. In this study, the cases requiring arbitration when the first reviewer and the second blinded reviewer disagreed were not evaluated by a third blinded arbitrator. Further studies are needed to overcome these limitations and confirm the findings of the present study.

## Conclusions

Ultrasonography within the first 6 months was necessary for management of children with SFU grade 1or 2 HN, because some patients showed worsening. After that, it is considered safe to stop close follow-up and spread the follow-up interval for stable cases. Most cases of perinatal SFU grade 1or 2 HN resolved spontaneously with conservative treatment. However, a few cases reappeared within 1 year. Therefore, ultrasonography after 1 year was necessary in children with HN that spontaneously disappeared. The appropriate time to end the follow-up was considered to have been after 1 year or more has passed since the disappearance was confirmed.

## Abbreviations

CI: Confidence interval
HN: Hydronephrosis
SFU: The Society of Fetal Urology

## References

[1] Livera LN, Brookfield DS, Egginton JA, Hawnaur JM (1989). Antenatal ultrasonography to detect fetal renal abnormalities: a prospective screening programme. BMJ.

[2] Blyth B, Snyder HM, Duckett JW (1993). Antenatal diagnosis and subsequent management of hydronephrosis. J Urol.

[3] Gunn TR, Mora JD, Pease P (1995). Antenatal diagnosis of urinary tract abnormalities by ultrasonography after 28 weeks’ gestation: incidence and outcome. Am J Obstet Gynecol.

[4] Scott JE, Wright B, Wilson G, Pearson IA, Matthews JN, Rose PG (1995). Measuring the fetal kidney with ultrasonography. Br J Urol.

[5] Sidhu G, Beyene J, Rosenblum ND (2006). Outcome of isolated antenatal hydronephrosis: a systematic review and meta-analysis. Pediatr Nephrol.

[6] Braga LH, McGrath M, Farrokhyar F, Jegatheeswaran K, Lorenzo AJ (2018). Society for Fetal Urology classification vs Urinary Tract Dilation grading system for prognostication in prenatal hydronephrosis: a time to resolution analysis. J Urol.

[7] Kohata E, Kimata T, Onuma C (2019). Natural course of isolated mild congenital hydronephrosis: a 2-year prospective study at a single center in Japan. Int J Urol.

[8] Madden-Fuentes RJ, McNamara ER, Nseyo U, Wiener JS, Routh JC, Ross SS (2014). Resolution rate of isolated low-grade hydronephrosis diagnosed within the first year of life. J Pediatr Urol.

[9] Rigas A, Karamanolakis D, Bogdanos I, Stefanidis A, Androulakakis PA (2003). Pelvi-ureteric junction obstruction by crossing renal vessels: clinical and imaging features. BJU Int.

[10] Dhillon HK (1998). Prenatally diagnosed hydronephrosis: the Great Ormond Street experience. Br J Urol.

[11] Stocks A, Douglas R, Frentzen B, Richard G (1996). Correlation of prenatal renal pelvic anteroposterior diameter with outcome in infancy. J Urol.

[12] Podevin G, Mandelbrot L, Vuillard E, Oury JF, Aigrain Y (1996). Outcome of urological abnormalities prenatally diagnosed by ultrasound. Fetal Diagn Ther.

[13] Nguyen HT, Herndon CD, Cooper C (2010). The Society for Fetal Urology consensus statement on the evaluation and management of antenatal hydronephrosis. J Pediatr Urol.

[14] Akhavan A, Schnorhavorian M, Garrison LP (2014). Resource utilization and costs associated with the diagnostic evaluation of nonrefluxing primary hydronephrosis in infants. J Urol.

[15] Fernbach SK, Maizels M, Conway JJ (1993). Ultrasound grading of hydronephrosis: introduction to the system used by the Society for Fetal Urology. Pediatr Radiol.

[16] Keays MA, Guerra LA, Mihill J (2008). Reliability assessment of Society for Fetal Urology ultrasound grading system for hydronephrosis. J Urol.

[17] Kohno M, Ogawa T, Kojima Y (2020). Pediatric congenital hydronephrosis (ureteropelvic junction obstruction): medical management guide. Int J Urol.

[18] Mallik M, Watson AR (2008). Antenatally detected urinary tract abnormalities: more detection but less action. Pediatr Nephrol.

[19] Koff SA (2000). Postnatal management of antenatal hydronephrosis using an observational approach. Urology.

[20] Noe HN, Magill HL (1987). Progression of mild ureteropelvic junction obstruction in infancy. Urology.

[21] Ericson BA, Maizels M, Shore RM (2007). Newborn society of fetal urology grade 3 hydronephrosis is equivalent to preserved percentage differential function. J Pediatr Urol.

[22] Zee RS, Herndon CDA, Cooper CS (2017). Time to resolution: a prospective evaluation from the Society for Fetal Urology hudronephrosis registry. J Pediatr Urol.

[23] Chertin B, Pollack A, Koulikov D, Rabinowittz R, Hain D, Hadas-Halpren I (2006). Conservative treatment of ureteropelvic junction obstruction in children with antenatal diagnosis of hydronephrosis: lessons learned after 16 years of follow-up. Eur Urol.

[24] Riccabona M, Avni FE, Blickman JG (2008). Imaging recommendations in paediatric uroradiology: minutes of the ESPR workgroup session on urinary tract infection, fetal hydronephrosis, urinary tract ultrasonography and voiding cystourethrography, Barcelona, Spain, June 2007. Pediatr Radiol.

[25] Gantti JM, Broecker BH, Scherz HC (2005). Antenatal hydronephrosis with postnatal resolution: how long are postnatal studies warranted?. Urology.

[26] Matui F, Shimada K, Matsumoto F (2008). Late recurrence of symptomatic hydronephrosis in patients with prenatally detected hydronephrosis and spontaneous improvement. J Urol.

[27] Alconcher LF, Tombesi MM (2012). Natural history of bilateral mild isolated antenatal hydronephrosis conservatively managed. Pediatr Nephrol.

[28] Nguyen HT, Benson CB, Bromley B (2014). Multidisciplinary consensus on the classification of prenatal and postnatal urinary tractdilation (UTD classification system). J Pediatr Urol.

[29] Chalmers DJ, Meyers ML, Brodie KE, Palmer C, Campbell JB (2016). Inter-rater reliability of APD, SFU and UTD grading systems in fetal sonography and MRI. J Pediatr Urol.

[30] Rickard M, Easterbrook B, Kim S (2017). Six of one, half a dozen of the other: Ameasure of multidisciplinary inter/intra-rater reliability of the society for fetal urology and urinary tract dilation grading systems for hydronephrosis. J Pediatr Urol.

